# Protistan-Bacterial Microbiota Exhibit Stronger Species Sorting and Greater Network Connectivity Offshore than Nearshore across a Coast-to-Basin Continuum

**DOI:** 10.1128/mSystems.00100-21

**Published:** 2021-10-12

**Authors:** Ping Sun, Xin Huang, Ying Wang, Bangqin Huang

**Affiliations:** a State Key Laboratory of Marine Environmental Science, Xiamen University, Xiamen, China; b Fujian Province Key Laboratory for Coastal Ecology and Environmental Studies, College of the Environment and Ecology, Xiamen University, Xiamen, China; Swansea University

**Keywords:** microeukaryote, prokaryote, community assembly, co-occurrence network, coast-to-basin continuum

## Abstract

Little is known regarding how community assembly and species association vary with habitat and depth. Here, we examined the assembly and association of protistan and bacterial communities across a coast-shelf-slope-basin gradient of the South China Sea using high-throughput sequencing of the V3 and V4 regions of the rRNA gene transcript. Our study revealed that homogenizing dispersal and drift exerted an influence on protistan communities comparable to that on bacterial communities. In contrast, selection and dispersal limitation exerted contrasting effects on the two microbial communities. Community assembly was governed to a greater degree by selection than by dispersal limitation in the bacterial community, and this was much lower in the protistan community. Moreover, this organismal assembly pattern was robust with habitat and depth. However, the relative importance of selection to dispersal limitation varied with habitat and depth in both communities, where horizontally it was higher offshore than nearshore and vertically it was lower in the bottom or deep chlorophyll maximum (DCM) than on the surface. The offshore possessed more microbial network complexity and more associations among microbial taxa than the nearshore, and vertically, the bottom possessed more complexity than the surface and the DCM. Moreover, temperature is strongly associated with the composition and co-occurrence of microbial communities, implying that temperature plays a dominant role in the selection of the protistan-bacterial microbiome across a coast-to-basin continuum. This study contributes to our understanding of the assembly mechanism and species association of protistan-bacterial microbiota across multiple habitats and depths.

**IMPORTANCE** Microbial organisms play a crucial role in global nutrient cycling. Few studies have attempted to simultaneously investigate the community assembly of microeukaryotes and prokaryotes and their association patterns in oceanic waters. This is especially true regarding how they vary with habitats and depths despite the fact that they are essential for developing a more holistic understanding of marine ecosystems. This study revealed the differential actions of selection and dispersal limitation and species association across a coast-to-basin continuum on the marine protistan-bacterial microbiome. Moreover, temperature was identified as a crucial factor driving the structure and co-occurrence of protistan and bacterial communities. The results emphasize that the differences in community assembly and association patterns between nearshore and offshore of the main constituents of the ocean microbiota should be considered to understand their current and future configurations. This is especially crucial in the context of climate change, as the response of ocean microbiota to nearshore and offshore temperature changes remains unknown.

## INTRODUCTION

Microbial communities are abundant and diverse and vary according to location and time (1). The spatiotemporal variability of the microbial community is shaped by a combination of deterministic and stochastic processes (2). Deterministic community assembly primarily results from selection, including environmental filtering and biotic interactions such as predation, mutualism, and parasitism (3). Stochastic community assembly occurs as a result of dispersal events such as dispersal limitation and homogenizing dispersal and demographic shifts in birth and death (4). Previous studies have examined the relative importance of deterministic and stochastic assembly processes on protistan and bacterial communities in a variety of aquatic environments (5–8). Bacterial communities were primarily structured by selection, whereas protistan communities were driven by drift/dispersal limitation in the eastern Antarctic coastal lakes (5) and the lakes of the Tibetan Plateau (8). Bacterial communities were shaped by the combination of selection, dispersal limitation, and drift, whereas picoeukaryotes were predominantly driven by dispersal limitation in the surface layer of the tropical and subtropical oceans (9). However, protistan communities were more structured by selection relative to dispersal limitation than bacterial communities in the East China Sea (6). The relative importance of deterministic and stochastic processes relies on spatiotemporal scales, habitat types, and species traits (10). To date, limited evidence exists regarding the assembly mechanisms of protistan and bacterial communities across habitats and depths. To the best of our knowledge, only one study indicated that the relative influence of selection to dispersal limitation was greater for protists in the bottom layer than in the surface/deep chlorophyll maximum (DCM) of the East China Sea (6). Assembly processes that shape marine protistan and bacterial communities have rarely been simultaneously investigated across a broad range of environmental gradients such as those that exist from the coast-to-basin continuum. Selection is likely to play an important role in dynamic habitats with pronounced environmental gradients such as those present in a coast-shelf-slope-basin continuum. Density differences among depth layers in the water column and water mass movements in oceanic waters can form barriers that prevent water mixing, thus leading to a crucial role of dispersal limitation. Drift is likely to play a less critical role in the assembly of large populations than in the assembly of small populations (10). Given the large population sizes of protists and bacteria in oceanic waters, selection and dispersal limitation may override the effects of drift on the assembly of the microbial communities. As a result, we expect selection and dispersal limitation to be two vital forces driving microbial community variability across a coast-to-basin continuum and water depths.

Protist-bacterium interactions are common in the ocean and are observed in the context of predator-prey relationships, symbiont/parasite-host relationships, and important substance exchanges such as the exchange of nutrients and vitamins (11, 12). Therefore, their associations undoubtedly play a crucial role in determining the community assembly of the protistan-bacterial microbiota (13). However, the two groups have rarely been investigated simultaneously as an organismal factor to reveal the role of biotic interactions in community assembly studies (9, 14). A recent study found that cross-domain organismic factors were essential variables for the assembly of protistan and bacterial communities in lakes and ponds throughout Europe (14). Therefore, we expect that protistan-bacterial interactions through the multiple, complex relationships mentioned above would result in a complex co-occurrence network. Co-occurrence network analysis has offered prominent information on the potential interactions between microbial organisms in a wide variety of environments such as soil (15), freshwater (16), and the ocean (17). Previous studies have demonstrated that the microbial co-occurrence network follows a nonrandom pattern and has a modular structure that is primarily determined by the taxonomic relatedness of the co-occurring species (18). Apart from ecological niche differences (niche-based theory), species co-occurrence may also be the result of dispersal and random processes of births and deaths (neutral theory) (19). Therefore, determining the relative importance of deterministic and stochastic processes in the assembly of microbial communities can shed light on the ecological strategies of co-occurring microorganisms (20).

Here, we examined the assembly and association of protistan and bacterial communities across spatial scales, i.e., horizontally from the coast to the basin and vertically from the surface to the bottom of the euphotic zone of the South China Sea (SCS), using high-throughput sequencing of the V3 and V4 regions of 16S and 18S rRNA gene transcripts, respectively (see Fig. S1A in the supplemental material). Specifically, we sought to address three questions of interest. (i) Protists have more complicated cell structures than bacteria, and they can exhibit a wide array of responses to environmental heterogeneity that may be less influenced by selection than bacteria. Based on this and the larger cell size of protists than of bacteria, we hypothesized that community assembly could be structured less by selection than by dispersal limitation in the protistan community, and this may occur to a much greater extent in the bacterial community. If this is the case, it would be interesting to determine how the relative importance of selection to dispersal limitation in regulating protistan and bacterial communities varies with habitats and depths. (ii) The SCS is one of the largest marginal seas of the Western Pacific Ocean (21). Our sampling area was influenced by both the Pearl River plume (PRP) and the internal tide that originated in the Luzon Strait and propagated westward into the SCS (22, 23). With increasing distance from the coast to the shelf, the Pearl River plume decreased its influence (Fig. S1B to I). Vertically, the internal tide reduced its impact from the bottom to the surface (Fig. S1J to Q). Surface water is generally less stratified than DCM and bottom water (Fig. S1B to Q). Therefore, dispersal limitation may result in a less critical role in the surface than in the other layers. We hypothesized that the intrusion of the Pearl River plume, the movement of the internal tide, and the stratification of the water layers would result in more stochastic effects on both microbial communities in the nearshore (coast and shelf) than in the offshore (slope and basin) and in the bottom and the DCM than in the surface. (iii) Additionally, given the ubiquitous association between protists and bacteria in the oceans, we hypothesized that multiple, complex relationships among protistan-bacterial microbiomes would result in complex co-occurrence networks in the present study. If this is the case, it would be interesting to examine how the protistan-bacterial microbiome co-occurrence differs between habitats and depths.

10.1128/mSystems.00100-21.1 FIG S1 (A) Map of sampling stations in the South China Sea. (B to Q) Hydrographic profiling showed temporal variation in the water column structures at the sampling sites, i.e., M11, M8, M4, and Seats. (B, F, J, and N) Temperature; (C, G, K, and O) salinity; (D, H, L, and P) density; (E, I, M, and Q) squared buoyancy frequency. The red solid lines in panels M and Q indicate the depth of maximum *N*^2^ in the offshore sampling sites (i.e., M4 and Seats). Download FIG S1, JPG file, 2.0 MB. Copyright © 2021 Sun et al. 2021 Sun et al. https://creativecommons.org/licenses/by/4.0/ This content is distributed under the terms of the Creative Commons Attribution 4.0 International license.

## RESULTS

### Community assembly of protistan-bacterial microbiota.

**FIG 1:**
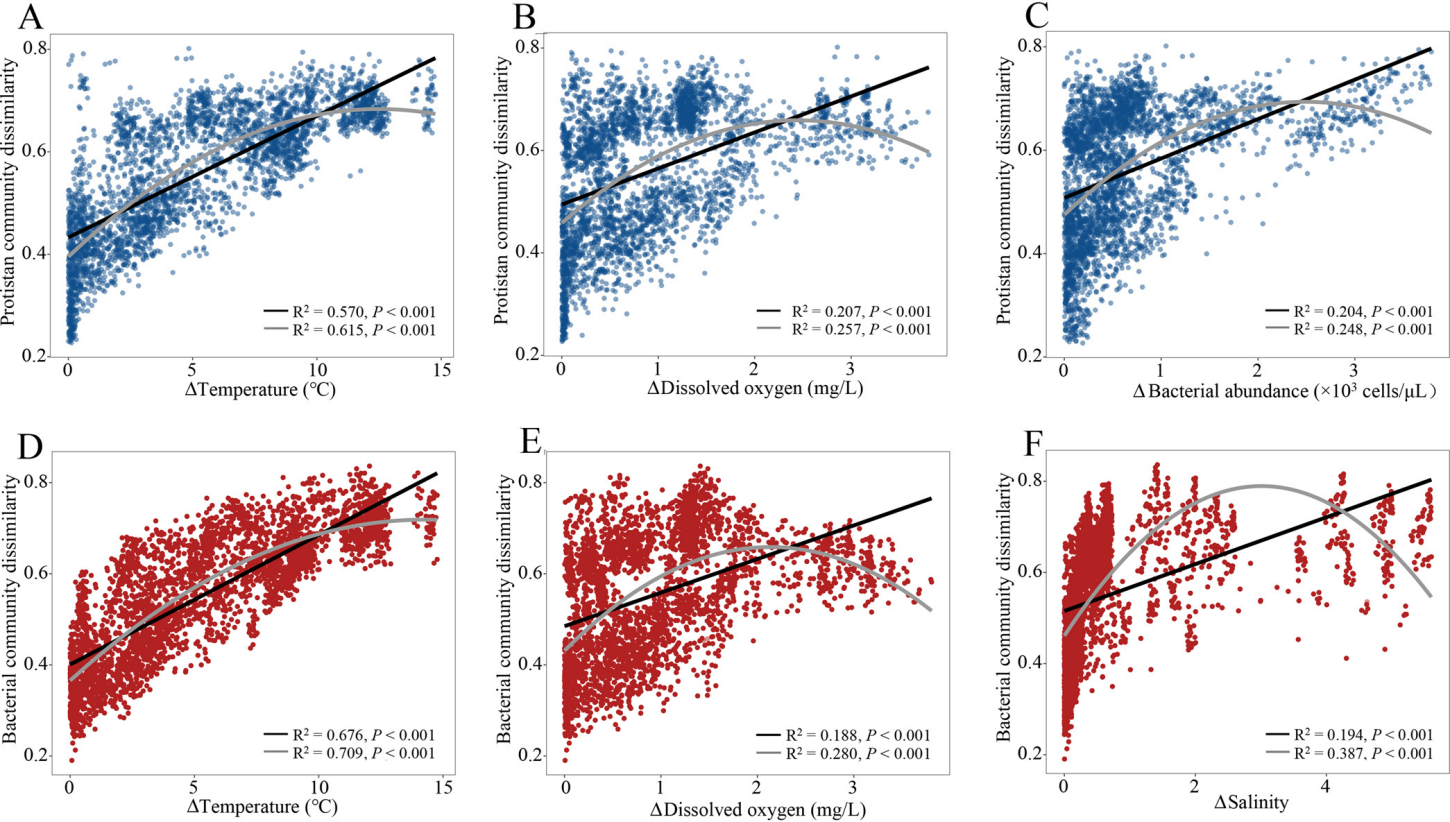
Correlations of protistan (A to C) and bacterial (D to F) community dissimilarities with differences in major environmental properties estimated by linear least-squares regression. First- and second-order polynomial fits are shown with black and gray solid lines, respectively.

In total, 6,198,192 high-quality reads were obtained from water samples across the coast-shelf-slope-basin continuum, and these were clustered into 6,532 protistan operational taxonomic units (OTUs) and 5,274 bacterial OTUs at a 97% similarity level (see Table S1 in the supplemental material). We used the phylogenetic null model to infer the underlying ecological processes that require a phylogenetic signal in habitat association. Habitat preferences of closely related taxa are more similar to each other than to the habitat preferences of distant relatives. First, we used multiple regression on distance matrices (MRM) analysis to identify the variables that were responsible for structuring differences in community compositions. The results revealed that temperature, dissolved oxygen (DO), and bacterial abundance were strong predictors of the dissimilarity of protistan communities in different habitats and at various water depths (Table S2). Temperature, dissolved oxygen, and salinity were also important factors responsible for driving the variation in bacterial communities (Table S2). Temperature was the most dominant factor for structuring the microbial community composition (Table S2). This result was confirmed by the finding that both protistan and bacterial community dissimilarity showed a significant positive relationship with the temperature difference (*R*^2^ = 0.57 and *P* < 0.001 for protists; *R*^2^ = 0.67 and *P* < 0.001 for bacteria), dissolved oxygen (*R*^2^ = 0.20 and *P* < 0.001 for protists; *R*^2^ = 0.18 and *P* < 0.001 for bacteria), bacterial abundance (*R*^2^ = 0.20 and *P* < 0.001 for protists), and salinity (*R*^2^ = 0.19 and *P* < 0.001 for bacteria) for pairwise comparisons (Fig. 1). This indicated that the more difference in temperature between the two samples, the more dissimilar the community composition between them. Additionally, temperature varies with depth, geographic distance, and other environmental factors, and based on this, a partial Mantel test was performed to estimate the effect of temperature on both communities after controlling for spatial distances and other environmental distances (Table 1). All the results strongly suggested that temperature was a crucial factor for structuring protistan and bacterial communities.

**TABLE 1:** Partial Mantel tests of protistan and bacterial community dissimilarities against distance matrix of temperature and other environmental and spatial variables

Bray-Curtis dissimilarity	Temperature.dist^*a*^ controlling for Env.dist^*b*^ (excluding temp) + Geo.dist^*c*^ + Depth.dist^*d*^	Env.dist (excluding temp) controlling for Temperature.dist + Geo.dist + Depth.dist	Geo.dist controlling for Env.dist (excluding temp) + Temperature.dist + Depth.dist	Depth.dist controlling for Env.dist (excluding temp) + Temperature.dist + Geo.dist
Protistan community				
*r*	0.465	0.429	0.288	−0.015
*P*	<0.001	<0.001	<0.001	0.599

Bacterial community				
*r*	0.527	0.423	0.427	0.116
*P*	<0.001	<0.001	<0.001	<0.001

a Temperature.dist, temperature dissimilarity based on Euclidean distance.

b Env.dist (excluding temperature), distance of all measured variables except temperature based on Euclidean distance.

c Geo.dist, geographic distance.

d Depth.dist, depth dissimilarity based on Euclidean distance.

10.1128/mSystems.00100-21.6 TABLE S1 Summary statistics of sequencing data in the present study. Download Table S1, DOCX file, 0.02 MB. Copyright © 2021 Sun et al. 2021 Sun et al. https://creativecommons.org/licenses/by/4.0/ This content is distributed under the terms of the Creative Commons Attribution 4.0 International license.

10.1128/mSystems.00100-21.7 TABLE S2 Multiple regression on dissimilarity matrices (MRM) results showed the percentage of variance in community composition of protists and bacteria explained by environmental variables. Values in boldface type indicate a significant correlation (*P* < 0.05). Ex.Var. (%), percentage of variance explained by environmental variables; Cum. (%), cumulative percentage of variance explained. Download Table S2, DOCX file, 0.02 MB. Copyright © 2021 Sun et al. 2021 Sun et al. https://creativecommons.org/licenses/by/4.0/ This content is distributed under the terms of the Creative Commons Attribution 4.0 International license.

To examine the phylogenetic signals, the top three variables identified by MRM analysis (temperature, DO, and bacterial abundance for the protistan community and temperature, DO, and salinity for the bacterial community) were included in the following phylogenetic signal analyses (Fig. S2). Mantel correlograms consistently revealed significant positive correlations across short phylogenetic distances for protistan and bacterial communities (*P* < 0.01) (Fig. S2). The phylogenetic distance across which there was a significant phylogenetic signal varied from 1.6% to 11.9% of the maximum phylogenetic distance within the phylogeny of each microbial group (Fig. S2). These results suggest that it would be appropriate to quantify phylogenetic turnover among the closest relatives using the phylogenetic null model.

10.1128/mSystems.00100-21.2 FIG S2 Phylogenetic signal tested using temperature, DO, and bacterial abundance/salinity for protists (A to C) and bacteria (D to F). The variables explain the highest fraction of the community variance of each of the two microbial groups. Solid dots show significant correlations (*P < *0.01) that indicate phylogenetic signals in species ecological niches, which were found across short phylogenetic distances for each of the two microbial groups. Download FIG S2, JPG file, 0.8 MB. Copyright © 2021 Sun et al. 2021 Sun et al. https://creativecommons.org/licenses/by/4.0/ This content is distributed under the terms of the Creative Commons Attribution 4.0 International license.

**FIG 2:**
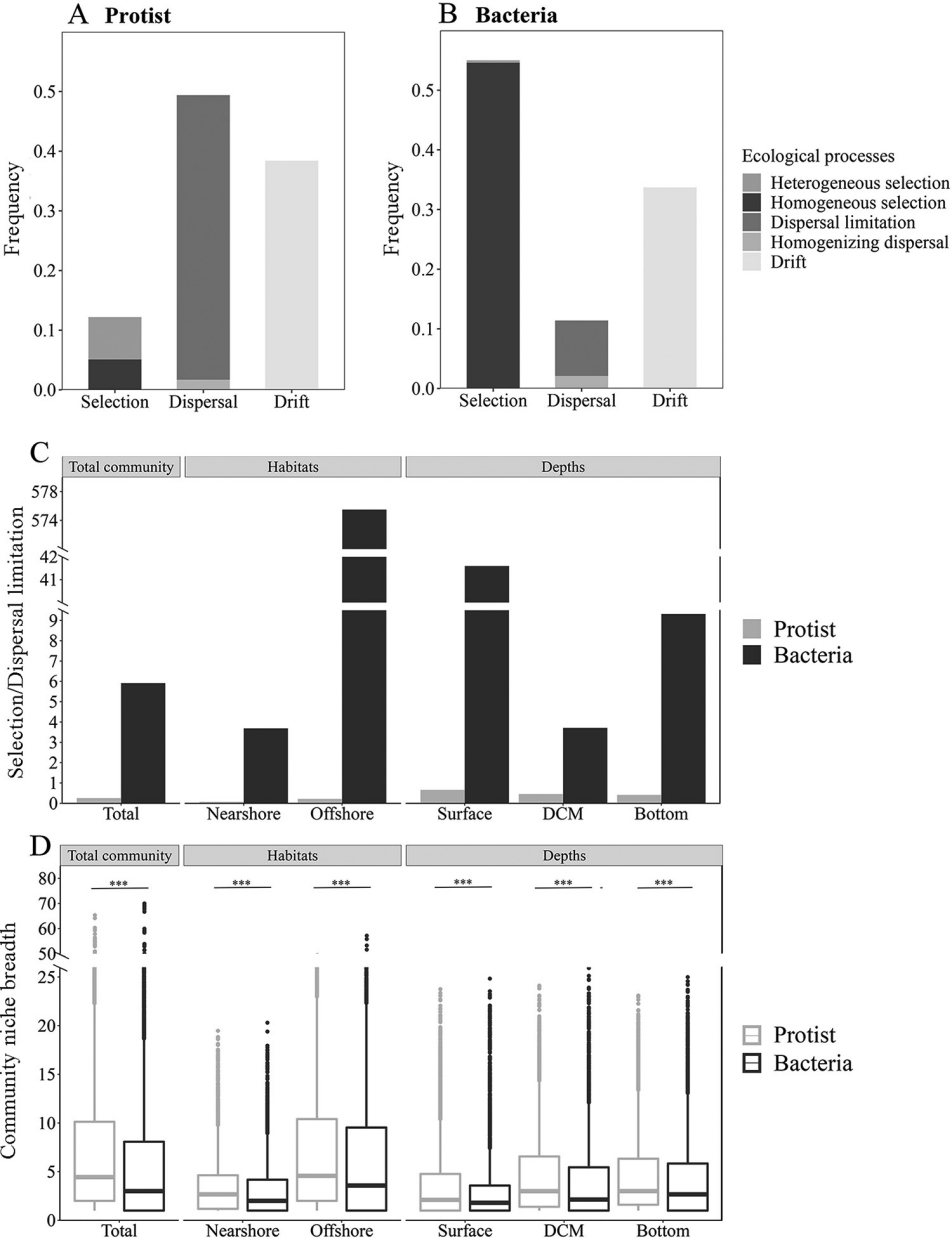
Community assembly (A to C) and niche breadth (D) of protistan-bacterial microbiota across habitats and depths. Selection and dispersal limitation were determined using βNTI, where phylogenetic distance is measured relative to a null model. Asterisks indicate a statistical difference in community niche breadth between protists and bacteria (***, *P* < 0.001 by a Wilcox rank-sum test).

The phylogenetic null model analyses revealed that homogenizing dispersal played a minor role in protistan and bacterial community assemblies that drove approximately 1.7 to 2.1% of the turnover in community compositions (Fig. 2A and B). Approximately 38.4% and 33.7% of the turnover in protistan and bacterial communities, respectively, were due to drift (Fig. 2A and B). Selection was the most critical process for structuring bacterial communities (55.0% of the overall community turnover), while dispersal limitation played a minor role in the community assembly (9.3% of the overall community turnover) (Fig. 2A and B). The contribution of selection and dispersal limitation exhibited a reverse trend in protistan community assembly (i.e., selection explained 12.2% of the overall community turnover, and dispersal limitation explained 47.4% of the overall community turnover) (Fig. 2A and B). The ratio of selection to dispersal limitation was higher in the bacterial community than in the protistan community, and this pattern was consistent across habitats and water depths (Fig. 2C). These results indicated that the bacterial community was more structured by selection relative to dispersal limitation than the protistan community. Variation partitioning analysis (VPA) showed similar results, where protists were less limited by selection relative to dispersal limitation than bacteria (Table S3). Additionally, both protistan (*R*^2^ = 0.6) and bacterial (*R*^2^ = 0.7) communities fit the neutral model (Fig. S3A and B). Approximately 56.3% and 68.7% of protistan and bacterial community variations, respectively, were explained by the neutral model (Fig. S3A and B). The migration rate (*m*) of the protistan community (0.06), as estimated by the neutral model, was lower than that of the bacterial community (0.09), thus suggesting that the protistan community was more limited by dispersal (Fig. S3A and B). To explain the relative contribution of selection and dispersal limitation to the assembly of protistan and bacterial communities, the habitat niche breadth at the community level (*B_com_*) was estimated. The *B_com_* for the protistan community was significantly higher than that for the bacterial community (Fig. 2D). Additionally, a similar tendency was seen both horizontally and vertically (Fig. 2D). Despite a consistent pattern of organismal assembly, the relative contribution of selection to dispersal limitation varied with distance in both microbial communities (Fig. 2C), being greater horizontally in the offshore than in the nearshore and being less vertically in the bottom/DCM than in the surface (Fig. 2C).

10.1128/mSystems.00100-21.3 FIG S3 Fit of the neutral community model (NCM) for protistan (A) and bacterial (B) OTUs. The solid blue lines indicate the best fit to the NCM, and the dashed blue lines represent 95% confidence intervals around the model prediction. OTUs that occur more or less frequently than predicted by the NCM are shown in different colors. R^2^ indicates the fit to the neutral model; m indicates the immigration rate. Download FIG S3, JPG file, 0.7 MB. Copyright © 2021 Sun et al. 2021 Sun et al. https://creativecommons.org/licenses/by/4.0/ This content is distributed under the terms of the Creative Commons Attribution 4.0 International license.

10.1128/mSystems.00100-21.8 TABLE S3 Variation partitioning results showing the contributions of environmental (E) and spatial (S) factors. Download Table S3, DOCX file, 0.02 MB. Copyright © 2021 Sun et al. 2021 Sun et al. https://creativecommons.org/licenses/by/4.0/ This content is distributed under the terms of the Creative Commons Attribution 4.0 International license.

### Co-occurrence networks of protistan-bacterial microbiota.

**FIG 3:**
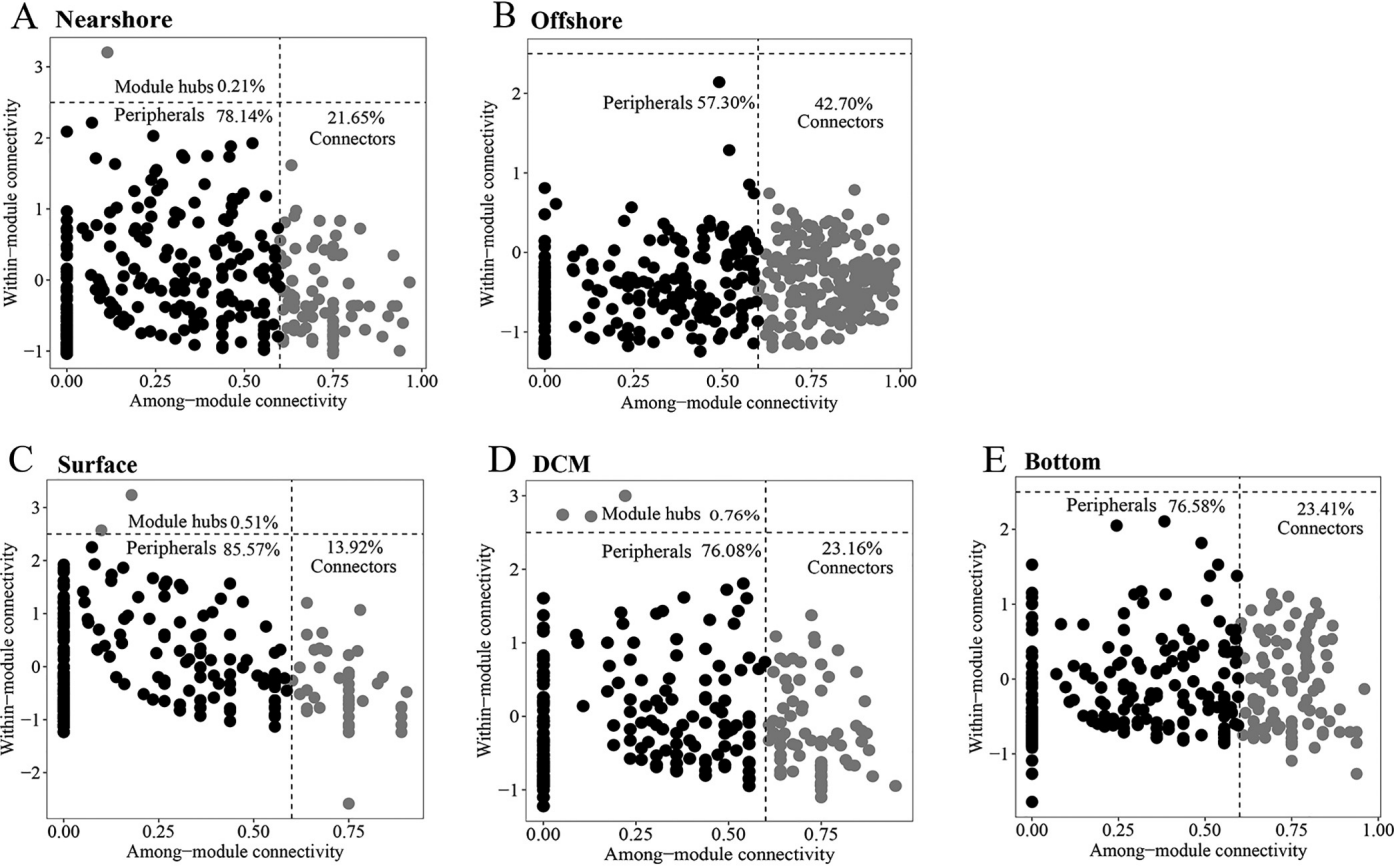
Z-C plot distribution of OTUs in co-occurrence networks across habitats (nearshore [A] and offshore [B]) and depths (surface [C], DCM [D], and bottom [E]).

Co-occurrence networks were constructed for the protistan-bacterial microbiota in the horizontal (nearshore-offshore) and vertical (surface-DCM-bottom) directions (Fig. S4). The percentage of negative correlation edges of the networks increased from the nearshore to the offshore and from the surface to the bottom, suggesting a clear spatial distribution pattern of the antagonistic relationship that may be due to resource availability (Fig. S4). We examined both OTU-level and network-level topological parameters, and our results revealed variations across habitats and depths (Fig. 3). We used the Z-C scatterplot to demonstrate the role of an OTU in a network in how a node is positioned within a specific module and how it interacts with other modules (Fig. 3). The results revealed that the proportion of connector nodes (that offered links among modules) increased from the nearshore (21.6%) to the offshore (42.7%); (Fig. 3A and B). In the vertical direction, the proportion of connectors increased from the surface (13.9%) to the bottom (23.4%) (Fig. 3C to E). However, no module hubs (that were highly connected within a module) were observed in the offshore and bottom networks (Fig. 3). Network hubs (that were highly connected, both within and between modules) were absent in all the networks. The above-described results indicated a less hub-based and more connected network structure for the offshore and the bottom. The network topological parameters, including average degree, average clustering coefficient, and network density, were higher in the offshore than in the nearshore and were higher in the bottom than in the surface and DCM, suggesting that microbial communities in the offshore and the bottom were more connected (Table 2). The average path length and network diameter were both lower in the offshore and bottom networks, indicating closer relationships among the microbial communities (Table 2).

**TABLE 2:** Topological features of co-occurrence networks of protistan-bacterial microbiota across habitats and depths

Location	Real networks							
	No. of nodes	No. of edges	Avg degree	Network diam	Network density	Modularity	Avg clustering coefficient	Avg path length
Nearshore	495	3,749	15.147	10	0.031	0.464	0.528	3.539
Offshore	566	14,398	50.876	13	0.090	0.238	0.668	2.791
Surface	415	2,020	9.735	10	0.024	0.533	0.517	3.833
DCM	413	1,981	9.593	11	0.023	0.493	0.482	3.831
Bottom	467	3,921	16.792	11	0.036	0.380	0.579	3.391

10.1128/mSystems.00100-21.4 FIG S4 Co-occurrence networks of protistan and bacterial OTUs across habitats (nearshore [A] and offshore [B]) and depths (surface [C], DCM [D], and bottom [E]). PPE, percentage of positive edges; PNE, percentage of negative edges. Only OTUs that are present in at least 60% of the samples were included in the analyses. Download FIG S4, JPG file, 2.9 MB. Copyright © 2021 Sun et al. 2021 Sun et al. https://creativecommons.org/licenses/by/4.0/ This content is distributed under the terms of the Creative Commons Attribution 4.0 International license.

**FIG 4:**
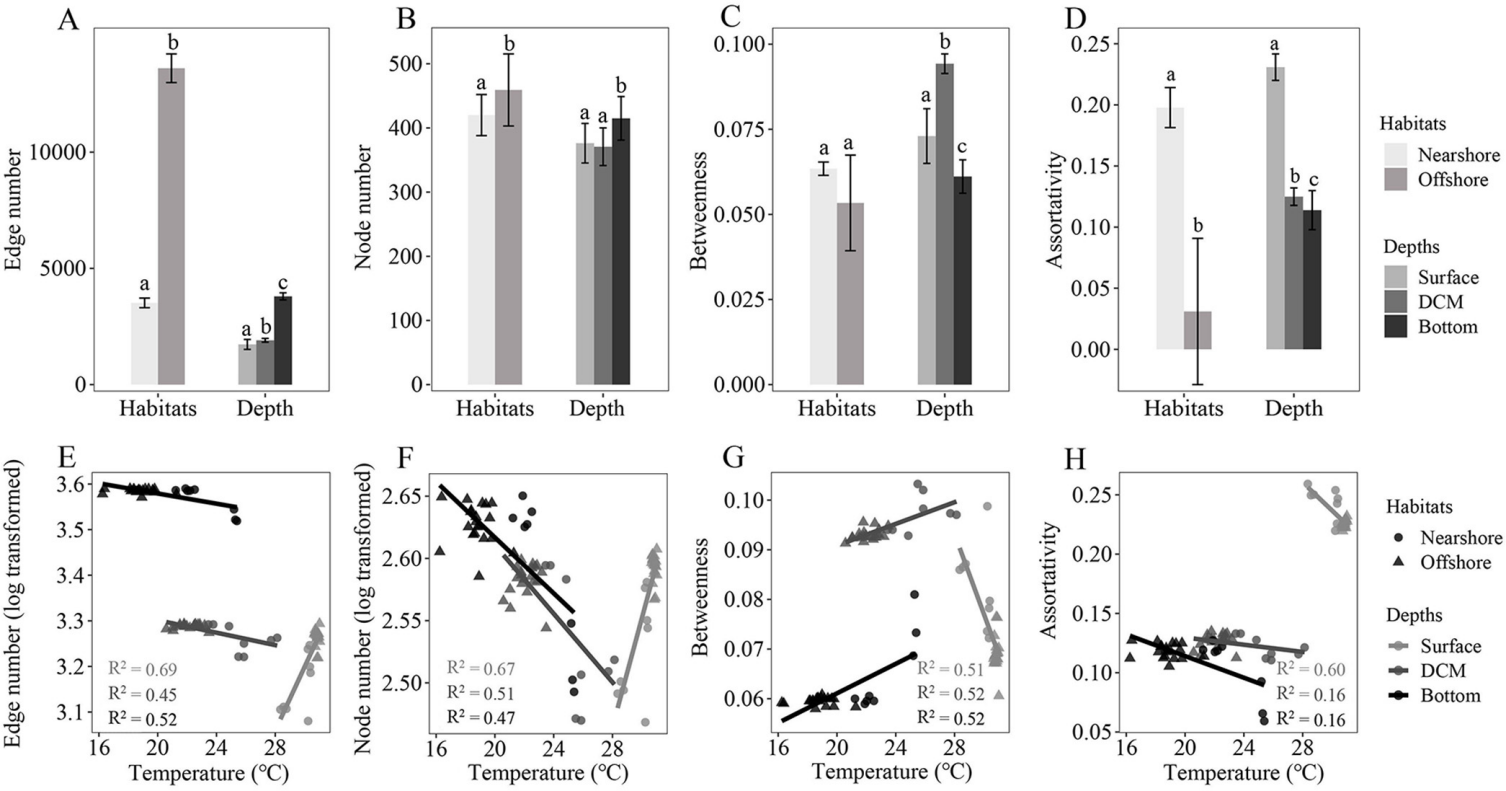
(A to D) Numbers of edges (A) and nodes (B) and degrees of betweenness (C) and assortativity (D) of co-occurrence patterns across habitats and depths. (E to H) Relationships of temperature to the numbers of edges (E) and nodes (F) and the degrees of betweenness (G) and assortativity (H) of co-occurrence patterns across depths (bars without shared letters indicate significant differences at a *P* value of 0.05 by a Wilcox rank sum test).

We assess the complexity of microbial networks using node numbers, edge numbers, betweenness, and assortativity (24). Networks exhibiting lower numbers of nodes and edges and higher betweenness and assortativity represented lower network complexity and vice versa (Fig. 4). The offshore and bottom networks had a greater number of nodes and edges and lower betweenness and assortativity, implying that they are more complex than the nearshore and the surface and DCM networks, respectively (Fig. 4A to D). Moreover, the number of nodes and edges increased and the betweenness and assortativity decreased in response to the increase of temperature in the surface layer, whereas the DCM and the bottom exhibited the reverse trend, with the exception that assortativity decreased with the increase of temperature (Fig. 4E to H). It is not surprising that the surface and the other two depth layers exhibit opposite trends. The temperature of the surface water increased from the nearshore to the offshore (see Materials and Methods for details), whereas the DCM and the bottom exhibited the reverse pattern. Based on this, contrasting results between depth layers were expected (Fig. S5). These findings suggested that temperature affected microbial associations and decreased the complexity of the microbial community networks as the temperature increased. Taken together, offshore and bottom protistan-bacterial microbiota networks exhibited greater connectivity and complexity, implying that species co-occurrence is more frequent in offshore communities than in nearshore communities and is more frequent in bottom communities than in surface and DCM communities, respectively.

10.1128/mSystems.00100-21.5 FIG S5 (A) Relationship between temperature and geographic distance at different sampling layers; (B and C) Profiles of temperature and salinity of the sampling transect from M11 to Seats. Download FIG S5, JPG file, 0.7 MB. Copyright © 2021 Sun et al. 2021 Sun et al. https://creativecommons.org/licenses/by/4.0/ This content is distributed under the terms of the Creative Commons Attribution 4.0 International license.

## DISCUSSION

### Assembly mechanisms of protistan-bacterial microbiota across habitats and depths.

The mechanisms underlying the community assembly of microorganisms have been studied extensively in recent decades (25). Here, we examined protistan and bacterial community assembly along a coast-shelf-slope-basin gradient in the photic zone of the South China Sea. Our study indicates that homogenizing dispersal and drift each had an influence on protistan communities comparable to that on bacterial communities, while selection and dispersal limitation had contrasting effects on the two microbial communities. The higher ratio of dispersal limitation to homogenizing dispersal of the protistan (28.1) than of the bacterial (4.4) communities suggested that protists were more constrained by dispersal limitation than bacteria, and this is in agreement with the size-dispersal hypothesis that the dispersal scales are negatively correlated with the cell size fraction of ocean plankton (26). The neutral model results also suggested that the migration rate of protists was lower than that of bacteria (see Fig. S3 in the supplemental material). Moreover, the protistan community exhibited wider niche breadths than the bacterial community across different habitats and water depths, indicating that the protistan community may be less influenced by the environment by exhibiting wider niche breadths (Fig. 2D). This finding is consistent with those of previous studies demonstrating that generalists exhibiting wider niche breadths tend to be influenced less by the environment, while specialists with narrower niche breadths exhibited the reverse trend (6, 9).

The neutral model revealed that protists were more limited by dispersal limitation than bacteria, and this is consistent with the phylogenetic null model. However, the neutral model implied that neutral processes contributed significantly to bacterial community variations, which contradicted the phylogenetic model’s results that selection accounted for nearly half of the overall community variations. This could be because the two approaches are fundamentally different (25). While the neutral model is a powerful tool for inferring the involvement of neutral processes in microbial communities, it is incapable of quantifying the contribution of deterministic processes to community assembly (25). The phylogenetic null model was constructed using heuristic randomization algorithms with no apparent biological mechanism. Because biotic factors are not necessarily clustered phylogenetically, the influence of selection is frequently underestimated (27). Furthermore, stochasticity (random fluctuation) does not imply neutrality (similar mean fitness across species). Additionally, a single process may contain stochastic and deterministic components. For instance, selection can be driven by environmental stochasticity, and dispersal limitation can be either deterministic, stochastic, or a combination of the two (25). As a result, comparing the results of the two approaches may be challenging. Future studies will be essential in elucidating the community assembly processes through the further development and integration of niche and neutral theories (25).

In this study, one of the objectives was to explore if and how the assembly mechanism of microbial communities varied with habitats and depths. Despite a consistent organismal assembly pattern, the relative importance of selection regarding dispersal limitation was different across habitats and depths, where horizontally the offshore was higher than the nearshore and vertically the bottom/DCM was lower than the surface (Fig. 2C). In the present study, the nearshore sampling sites were influenced by the Pearl River plume, particularly at site M11 (Fig. S2) (22). The movement of water mass could facilitate high dispersal rates of microbes that inhabit them while strongly limiting dispersal among water masses. Therefore, the intrusion of the Pearl River plume could generate greater dispersal limitation effects on the nearshore than on the offshore communities. The South China Sea is well known for the presence of internal tides (28). The ridges of the Luzon Strait are the primary sources of internal tides in this region. Internal tides formed in the Luzon Strait radiate westward into the SCS and eastward into the Western Pacific Ocean (29). The propagation of internal tides in the northern part of the SCS exhibits a considerable coverage distance (over 500 km) and crosses over the deep basin and steep or gentle continental slope and shelf (Fig. S3) (30). The oscillation of the internal tide can induce strong vertical movement of water, thus causing the microorganisms that inhabit these waters to experience the up-and-down fluctuations in the lower water columns. Unlike zooplankton, the dispersal of ocean microbiota is more highly influenced by water physical movement due to the limited mobility of the latter (1). Therefore, we expected that the impact of dispersal limitation on nonsurface microbial communities would be greater than that on the surface communities. Additionally, wind can facilitate the dispersal of microorganisms, which decreases its impact from shallow to deep waters (31, 32). Moreover, surface water generally exhibited a lower stratification level than the DCM and bottom layers (Fig. S1), which can generate a less critical role of dispersal limitation in the surface than in the other layers. Dispersal limitation was therefore expected to have less of an influence on the surface microbial communities than on those below the surface. This is in contrast to the finding in the East China Sea that protists were increasingly influenced by selection relative to dispersal limitation as the water depth increased (6). This discrepancy could be partially due to differences between sampling areas. The East China Sea is primarily shallow, and approximately three-fourths of the sea is less than 200 m in depth (33). In a study by Wu et al. (6), the bottom samples were collected from sampling sites possessing water depths of less than 104 m, which were located close to the sediments (<7 m). Therefore, the bottom community could be strongly influenced by the hydrographic conditions of sediments at the bottom (for example, DO and NO_2_, as stated in the study), and this may have increased the relative importance of selection in regard to dispersal limitation (6).

Given the large population size of microorganisms residing in marine waters, it is surprising to see that drift explained approximately one-third of the protistan and bacterial community variations. The present study focused on a spatial scale of approximately 535 km, and this is a very small spatial scale compared to that of the global oceans. Moreover, drift was estimated via community snapshots taken from complex natural microbial communities. Therefore, a small community size may increase the relative influence of drift, even if the absolute community size is large (34). There are reports that drift plays a moderate role in the assembly of picoeukaryotes and prokaryotes over large spatial scales such as those present in tropical and subtropical surface oceans (9). A possible reason could be that other random processes resemble drift in large microbial populations. As stated previously by Logares et al. (9), the arrival of a new bacteriophage may attack abundant bacteria, and this could randomly reshuffle the local species abundances.

### Co-occurrence of protistan-bacterial microbiota across habitats and depths.

Despite the ubiquitous associations between protists and bacteria, few studies have considered the protistan community as a factor shaping bacterial community assembly and vice versa (14, 35). In this study, we examined the co-occurrence of protistan and bacterial communities across habitats and depths. We observed that the offshore co-occurrence network possessed a greater number of negative correlations than the nearshore network, suggesting higher rates of potentially antagonistic interactions among taxa offshore than nearshore (Fig. S4). It has been estimated that 300 to 380 Tg of organic carbon per year, 37 to 66 Tg of total nitrogen per year, and 4 to 11 Tg of total phosphorus are transferred from rivers and continents to coastal regions via river plumes (36, 37). The nearshore therefore possesses a more enriched nutrient resource pool than the offshore. Greater resource availability is speculated to reduce antagonistic relationships such as competition in microbial communities (38). It is therefore likely that nutrient input by river plumes in coastal regions contributes to the reduction of antagonistic relationships. In the vertical direction, unlike the DCM, where both light and nutrients are ideal for microorganisms to grow, the bottom layer is nutrient rich but light deficient (39), and this may result in a higher level of competitive exclusion of some closely related taxa. Therefore, the bottom co-occurrence network is expected to possess a more negative correlation than the DCM.

Understanding community assembly and species coexistence is essential for the study of microbial ecology. The theoretical framework of community assembly and species coexistence is a synthesis of environmental filtering and contemporary coexistence theory (40). The concept of environmental filtering is rooted in the assembly study of the plant community and outlines how the environment functions as a filter, allowing species from the regional pool with certain traits to become established in local communities (41). Coexistence theory in the modern era emphasizes coexistence on a local scale and incorporates niche differences and fitness similarities (42). While species coexistence strategies have been extensively investigated in plant communities, they have received little attention in microbial communities (20, 43). In the present study, species co-occurrence increased from the nearshore to the offshore as the relative influence of selection to dispersal limitation increased in the assembly of microbial communities (Fig. 2 and 3). Our findings corroborated those of another study on the bacterial community in undisturbed aquifers, which indicated that more interconnected bacterial communities were more susceptible to selection and had lower community turnover rates (44). The present study found a significant correlation of the composition and co-occurrence of protistan and bacterial communities with water temperature (Table 1 and Fig. 1 and 4), indicating the critical role of temperature in shaping the composition and co-occurrence of protistan and bacterial communities across the coast-to-basin continuum. Consistent with our findings, previous research has established that temperature is a crucial determinant of the structure of microbial communities in oceanic waters, demonstrating the critical role of temperature at both local and global scales (9, 45, 46). This could be partly explained by the physiological properties of bacteria and protists as well as the trophic interactions that exist between microbial organisms. Temperature is known to be a critical component in the growth of bacteria and protists (47, 48). Additionally, temperature may have an effect on the abundance of protist prey and/or parasites/symbionts and vice versa (49–51). As a result, we hypothesized that water temperature could influence the composition and co-occurrence of protistan and bacterial communities in two ways: (i) directly affecting the growth of protists and bacteria and (ii) indirectly shaping protistan and bacterial communities through interactions of prey-predator and/or parasite/symbiont-host. Taken together, our findings suggest that water temperature plays a critical role in the selection of the protistan-bacterial microbiota across the coastal-to-basin continuum.

### Conclusions.

In this study, we examined the community assembly and species co-occurrence of protistan-bacterial microbiota across the coast-to-basin continuum in the photic zone of the South China Sea. Homogenizing dispersal and drift had an influence on protistan communities comparable to that on bacterial communities, while selection and dispersal limitation had contrasting effects on the two microbial communities. Bacteria were more subject to selection relative to dispersal limitation than protists, and this pattern was robust across habitats and depths. Offshore communities were more governed by selection relative to dispersal limitation than were nearshore communities, while bottom/DCM communities were less governed than the surface communities. Offshore communities were more governed by selection relative to dispersal limitation than nearshore communities, while bottom/DCM communities were less governed than the surface communities. Microbial co-occurrence networks exhibited more connected and more complicated structures horizontally offshore than nearshore and vertically at the bottom than at the surface and DCM. Moreover, temperature was identified as a crucial factor shaping the composition and co-occurrence of both protistan and bacterial communities, which may provide a better understanding of the adaptation of microbial communities to environmental changes such as climate change. This study expanded the knowledge of the assembly and co-occurrence of protistan-bacterial microbiota across habitats and depths and revealed the crucial role of temperature in selection.

## MATERIALS AND METHODS

### Sampling.

Samples were collected from the coastal region to the basin of the northern South China Sea (NSCS) along a transect from 39 to 535 km off the coast of Guangdong Province from 20 to 29 June 2019, onboard the R/V *Tan Kah Kee* (TKK) (see Fig. S1A in the supplemental material). The choice of the transect was based on the previous characterization of the area in the context of a routine monitoring project of the NSCS. This project is funded by the Natural Science Foundation and conducts cruises each year. The investigated transect, which we studied in this study, covers the typical habitats of the NSCS. The transect crosses the coast (site M11, water depth of 23 m), shelf (site M8, water depth of 91 m), slope (site M4, water depth of 1,645 m), and basin (site Seats, water depth of 3,907 m) in the northern SCS (Table S4). Hydrographic profiling revealed temporal variations in the water column structures at the sampling sites (Fig. S1). For the nearshore sampling sites M11 and M8, the upper water column was occupied by warm, less saline water sourced from the Pearl River plume, indicating the intrusion of the PRP into the coastal and shelf regions (Fig. S1B to I). For the offshore sampling sites M4 and Seats, the evident synchrony of the up-and-down fluctuations of the isotherms (Fig. S1J and N), isohalines (Fig. S1K and O), and pycnoclines (Fig. S1L and P) indicated internal tidal oscillations. Moreover, the squared buoyancy frequency (*N*^2^) profiles (Fig. S1M and Q) also demonstrated that the variations of the most intense stratification and the depth of the highest *N*^2^ coincided with fluctuations of isotherms, isohalines, and pycnoclines, revealing the influence of internal tides on the slope and basin regions (Fig. S1). The surface temperature was increased from the nearshore to the offshore in the present study, and this was the opposite of that observed for the deep chlorophyll maximum (DCM) and the bottom (Fig. S5A). The profile of temperature and salinity of the transect revealed that sampling site M11 was influenced by intensified upwelling over the widened shelf in the northeastern South China Sea that lowered the surface temperature (Fig. S1B and C and Fig. S5B) (52). Additionally, the samples were collected from offshore to nearshore (from Seats to M11) (Table S4). Nearshore sampling was subjected to the influence of Typhoon Mun (4 July 2019) that resulted in a decreased surface temperature. Collectively, the surface temperature from the nearshore to the offshore exhibited a reverse trend from that of the DCM and bottom layers due to the intensified upwelling and the influence of Typhoon Mun. Considering the possible diurnal and depth differences in the microbial communities, we collected samples diurnally from three water depths that included the surface (5 m), the DCM, and the bottom of the euphotic zone (Table S4). A total of 84 water samples were collected (Table S4). Approximately 15 liters of 200-μm-prefiltered seawater was sampled using Niskin bottles attached to a conductivity-temperature-depth (CTD) profiler (SBE 917) equipped with a probe for conductivity, temperature, and salinity. The collected seawater was then filtered through a 0.22-μm-pore-size polycarbonate filter (Millipore, USA) using a peristaltic pump. The filters were flash-frozen and stored at −80°C until RNA extraction.

10.1128/mSystems.00100-21.9 TABLE S4 Sampling information for waters collected from the South China Sea. Download Table S4, DOCX file, 0.03 MB. Copyright © 2021 Sun et al. 2021 Sun et al. https://creativecommons.org/licenses/by/4.0/ This content is distributed under the terms of the Creative Commons Attribution 4.0 International license.

Hydrodynamic profiles for depth, temperature, and salinity were measured using a CTD probe. For bacterial and viral abundance analyses, 1.8 ml of a 20-μm-prefiltered sample was fixed with ice-cold glutaraldehyde at a final concentration of 1% for 15 min in the dark, flash-frozen in liquid nitrogen, and stored at −80°C. Bacterial and viral abundances were analyzed using a flow cytometer (Epics Altra II; Beckman Coulter). For photosynthetic pigment analysis, 6 to 8 liters of seawater samples was filtered through Whatman GF/F filters and stored at −80°C for later analysis. Pigment extraction was performed as described previously by Huang et al. (53). Briefly, pigments were extracted using 2 ml of *N*,*N*‐dimethylformamide in a freezer (−20°C) for 2 h. Prior to analysis, extracts were cleared by filtration through 13-mm Whatman GF/F filters to remove filter debris before mixing with a 1-mol/liter ammonium acetate solution (600 μl–600 μl). Photosynthetic pigment concentrations were measured using high-performance liquid chromatography (HPLC) (UltiMate 3000; Thermo). The chlorophyll *a* (Chl *a*) concentration was derived from the pigment analysis. The CHEMTAX program was used to determine the group composition of phytoplankton (54). Three pigment-based ratios were estimated as proxies for the relative abundances of micro-, nano-, and picometer-sized phytoplankton. The definitions of ratios for phytoplankton functional groups followed those described previously by Vidussi et al. (55). Samples for nanoflagellate enumeration were prefiltered through a 20-μm nylon mesh (Millipore, USA) to exclude larger plankton. Approximately 50 ml of seawater was preserved with glutaraldehyde (at a final concentration of 0.5%) at 4°C for 15 min, filtered onto black 0.8-mm polycarbonate filters with approximately 1 ml of liquid remaining, and stained with DAPI (4′,6-diamidino-2-phenylindole; Sigma, USA) for 10 min, and all the liquids were filtered out. The filters were sealed with paraffin onto microscope slides and stored at −20°C. Nanoflagellates were enumerated using fluorescence microscopy (BX51; Olympus, Japan) (56, 57).

### Extraction and PCR.

Polycarbonate filters were cut into small pieces and subjected to bead beating to allow mechanical lysis. A commercial extraction kit, the RNeasy minikit (Qiagen, USA), was used for RNA extraction. All extraction steps were performed according to the manufacturer’s instructions. The RNase-free DNase set (Qiagen, Germany) was used to remove the remaining DNA from the RNA extracts. RNA was immediately reverse transcribed to cDNA using a high-capacity cDNA reverse transcription kit (Applied Biosystems, USA) as described by the kit protocol. Primers 341F (5′-CCTAYGGGRBGCASCAG-3′) and 806R (5′-GGACTACNNGGGTATCTAAT-3′) were used to amplify the V3-V4 region of the 16S rRNA gene transcript (58). The protistan community was profiled by targeting the V4 region of the 18S rRNA gene transcript using the eukaryote-specific primers TAReuk454FWD1 (5′‐CCAGCASCYGCGGTAATTCC‐3′) and TAReukREV3 (5′‐ACTTTCGTTCTTGATYRA‐3′) (59). PCR conditions were described previously by Roggenbuck et al. (58) and Stoeck et al. (59). Each sample was amplified in triplicate, pooled, and purified using the Wizard SV gel and PCR cleanup system kit (Promega, USA). Paired-end sequencing of the amplicons from cDNA templates was performed using the Illumina MiSeq platform by Meiji Bioinformatics Technology Co. Ltd. (Shanghai, China).

### Sequence processing.

Illumina reads of both 16S and 18S rRNA gene transcripts were quality checked using Trimmomatic (60) and Flash (61). The criteria were as follows: (i) low-quality reads with an average quality score of <20 and a read length of <50 bp were discarded, (ii) reads with ambiguous characters and mismatches in barcodes or primers were removed, and (iii) reads that exhibited overlapping regions that were <10 bp or had a mismatch ratio of >0.2 were discarded. After quality control, chimera detection was performed with UCHIME (62) using the Protist Ribosomal Reference database v4.11.1 (PR2) (63) and SILVA v132 (64) as the reference databases. The singletons were removed to avoid the risk of sequencing errors. After removing chimeras and singletons, the final curated reads were clustered into operational taxonomic units (OTUs) using USEARCH v10 (65) with 97% sequence similarity (9). Representative reads of each OTU were taxonomically classified against PR2 v4.11.1 and SILVA v132 by BLAST. After taxonomic assignment, nonprotist and nonbacterial OTUs were removed. To normalize the sampling effort, the OTU reads for each sample were rarefied to 32,226 reads for the bacterial community and 41,562 reads for the protistan community for further analyses; these corresponded to a minimum number of reads per sample for bacterial and protistan communities. Phylogenetic trees were constructed for both groups (protists and bacteria) as described previously by Logares et al. (9). Briefly, OTU-representative sequences were aligned with Mothur against an aligned template from SILVA (66). TrimAl was then used to remove the poorly aligned sequences/nucleotides (67). Phylogenetic trees were constructed using FastTree implemented in QIIME (68).

### Phylogenetic null model analysis.

To infer ecological processes, the phylogenetic signal across phylogenetic distances was first examined according to the method of Stegen et al. (69). Temperature, DO, bacterial abundance, and salinity (these are the top environmental variables identified by MRM that explained a large percentage of the variance in the community composition of protists and bacteria, as described in Table S2) were selected as environmental variables for niche differences (Fig. S2). Mantel correlograms with 999 randomizations for significance tests were performed using Vegan (http://vegan.r-forge.r-project.org). Phylogenetic null model analysis was then performed according to the method of Stegen et al. (70) to quantify community assembly processes into the underlying driving forces of dispersal limitation, selection, and drift. First, the weighted β-mean nearest taxon distance (βMNTD) was calculated to estimate phylogenetic turnover, quantifying the mean phylogenetic distance between the evolutionarily closest OTUs in the two communities. The null model expectation was generated using 1,000 randomizations. Next, the β-nearest taxon index (βNTI) was calculated to determine the deviation of the observed βMNTD from the null βMNTD, suggesting whether selection or dispersal/drift structured community composition. Raup-Crick measurements using Bray-Curtis dissimilarities (RC_BC_) were calculated to differentiate between dispersal limitation and homogenizing dispersal. Randomization was performed 1,000 times. Next, the RC_BC_ metric was calculated to determine the deviation of the observed Bray-Curtis dissimilarities from the null Bray-Curtis values, which provided insight into the contribution of dispersal events (i.e., dispersal limitation and homogeneous dispersal) to community assembly (70). To reveal the environmental factors influencing community phylogenetic turnover, standard and partial Mantel tests were performed to test the statistical significance of the βNTI of microbial communities and environmental factors.

### Variation partitioning analysis and neutral community model.

We also employed variation partitioning analysis (VPA) and the neutral community model (NCM) to infer the ecological processes responsible for shaping microbial communities (71, 72). To determine environmental heterogeneity, we performed a principal-component analysis (PCA) using the R package Vegan v.2.5-7 (73). Next, the axes were selected according to the Kaiser-Guttman rule. To detect the spatial pattern, we accomplished this using distance-based Moran eigenvector maps (MEMs) in the R package adespatial v.0.3-14 (74). Finally, canonical correlation analysis (CCA) was performed to determine the relative effects of the environmental and spatial factors on community variations using Vegan v.2.5-7 (73). We used the NCM to estimate the potential contribution of neutral processes to microbial community assembly (72). Sloan’s NCM is based on Hubbell’s model of the neutral theory of biodiversity but is applicable to microbial communities (19, 72). The NCM predicts the relationship between the frequency with which taxa occur in a set of local communities and their abundance across the wider metacommunity (72). Under neutral community assembly, highly abundant taxa should be widespread since they are more likely to disperse by chance among different sampling sites, whereas rare taxa should be more likely to be lost in different sites due to ecological drift. For model fitting, we followed the approach used by Burns et al. (75). *R*^2^ represents the goodness of fit for the NCM. The parameter *m* represents the estimated migration rate, and lower *m* values indicate that microbial communities are more dispersal limited. Calculation of 95% confidence intervals around all fitting statistics was done by bootstrapping with 1,000 bootstrap replicates.

### Community niche breadth.

To help reveal the patterns of species sorting to dispersal limitation and the influence on protistan and bacterial communities, Levins’ niche breadth (*B*) index was calculated using the “niche.width” function in the package spaa (76). The formula is as follows:

Bj=1∑i=1NPij2

where *B_j_* represents the habitat niche breadth of OTU *j* in a metacommunity, *N* is the total number of communities in each metacommunity, and *P_ij_* is the proportion of OTU *j* in community *i*. The average *B* values of all taxa in a single community (*B_com_*) were calculated as an indicator of habitat niche breadth at the community level.

### Network analysis.

To estimate the species coexistence of the protistan-bacterial microbiota, co-occurrence networks across habitats (nearshore-offshore) and depths (surface-DCM-bottom) were constructed with an abundance-based OTU table. To reduce network complexity, only OTUs occurring in more than 60% of the samples were retained for the subsequent analysis. SparCC was used to infer pairwise correlations between OTUs (77). All *P* values were adjusted for multiple testing using the Benjamini-Hochberg false discovery rate (FDR)-controlling procedure with the R package multtest (78). Next, significant (*P* value of <0.01) and robust (|*r*| ≥ 0.7) correlations between OTUs were exported as a GML format network file. To balance the unequal sampling efforts between habitats (nearshore versus offshore), randomly selected same-size sample sets were included in the analysis (24 nearshore samples and 24 offshore samples). Network visualization and node-level topological properties were performed using Gephi v0.9.2 and Cytoscape v3.7.2. (79, 80). To differentiate their roles in the network, i.e., how a node is positioned within a specific module and how it interacts with other modules, the nodes were classified into four categories (i.e., network hubs, module hubs, connectors, and peripherals) (81). The characterization of node categories was based on their within-module degree (i.e., z-score) and among-module connectivity values (i.e., c-score, which is equivalent to the participation coefficient, i.e., *P_i_* as described by Poudel et al. and Guimerà and Amaral [81, 82]). Nodes with z-scores of >2.5 and c-scores of >0.6 were defined as network hubs that were highly connected, both within and between modules. Those with z-scores of >2.5 and c-scores of <0.6 were defined as module hubs that were highly connected within a module. Nodes with z-scores of <2.5 and c-scores of >0.6 were connectors that offered links among modules. Nodes with z-scores of <2.5 and c-scores of <0.6 were peripherals that provided few links with other nodes (81). The z-score and c-score were calculated using the Cytoscape plug-in GIANT (83), according to the methods described previously by Guimerà and Amaral (82). The formulas are as follows:

$$Z_{i}=\frac{k_{\mathrm{ib}}-{\bar{k}}_{b}}{{\text{σ}}_{k_{b}}}$$

and

$$P_{i}=1-\overset{N_{M}}{\underset{c=1}{\sum }}{\left(\frac{k_{\mathrm{ic}}}{k_{i}}\right)}^{2}$$

where *k_ib_* is the number of links of node *i* with other nodes in its module; ${\bar{k}}_{b}$ and ${\text{σ}}_{k_{b}}$ are the average value and the standard deviation of within-module connectivity over all the nodes in module *b*, respectively; *k_i_* is the number of links of node *i* in the whole network; *k_ic_* is the number of links from node *i* to nodes in module *c*; and *N_M_* is the number of modules in the network.

### Data availability.

All the sequences for 16S and 18S rRNA gene transcripts from this study have been deposited in the public NCBI Sequence Read Archive (SRA) database under BioProject accession number PRJNA687998.
