# Supplementary figures and images for: Protistan-Bacterial Microbiota Exhibit Stronger Species Sorting and Greater Network Connectivity Offshore than Nearshore across a Coast-to-Basin Continuum
Source: mSystems. 2021 Oct 12;6(5):e00100-21. doi: 10.1128/mSystems.00100-21 (PMC8510552; doi:10.1128/mSystems.00100-21)

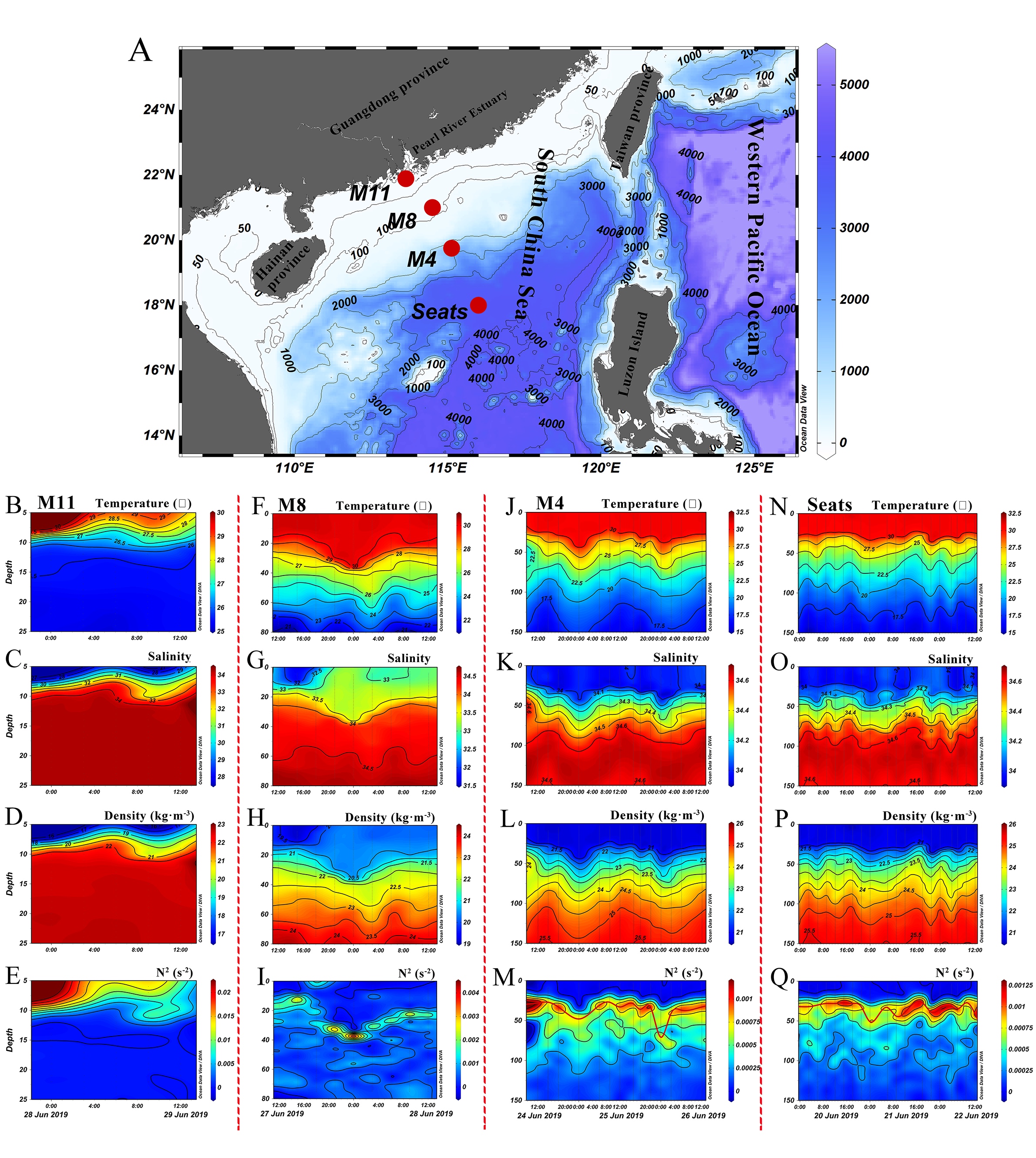

Supplement: FIG S1 [file msystems.00100-21-sf001.jpg]

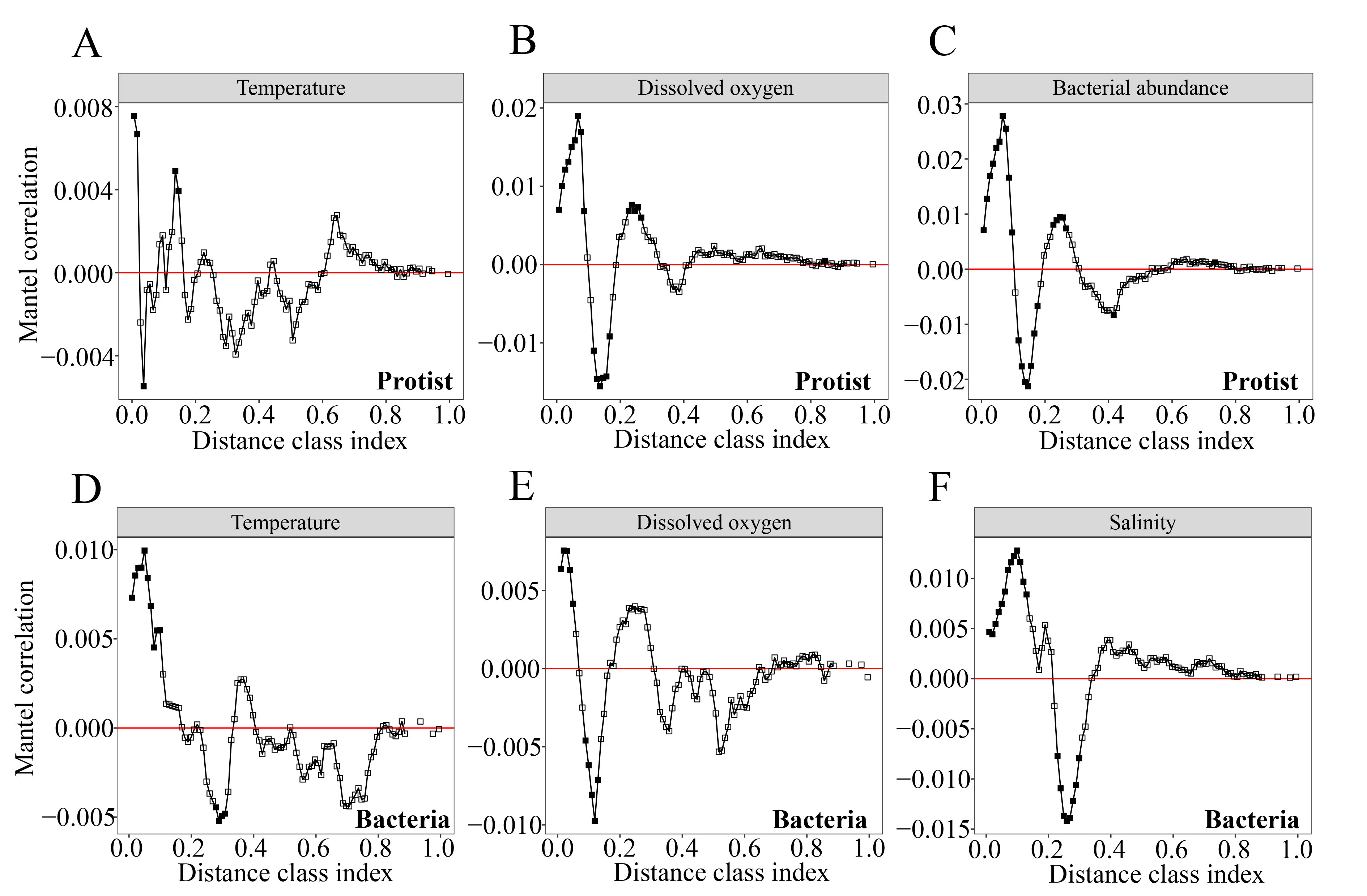

Supplement: FIG S2 [file msystems.00100-21-sf002.jpg]

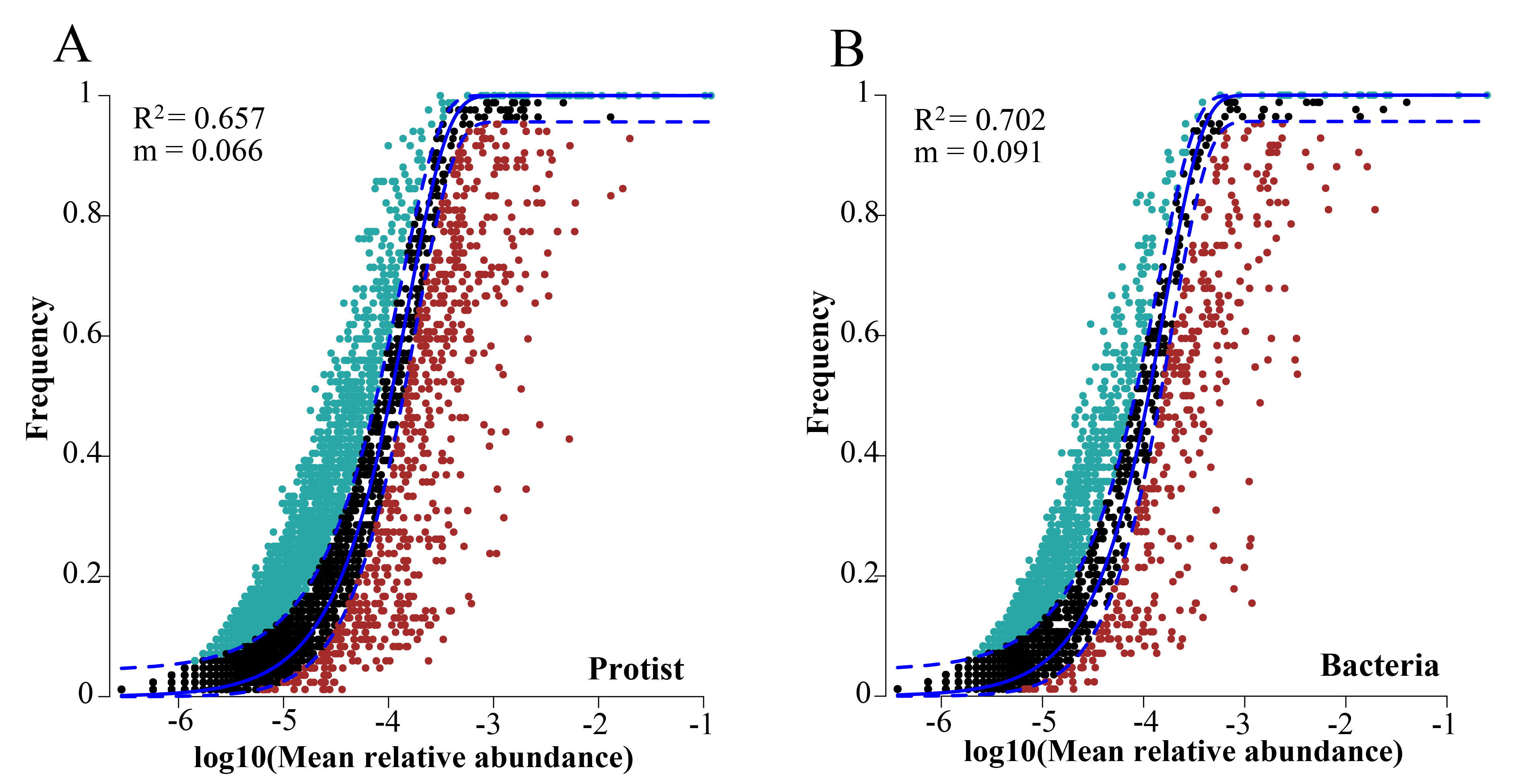

Supplement: FIG S3 [file msystems.00100-21-sf003.jpg]

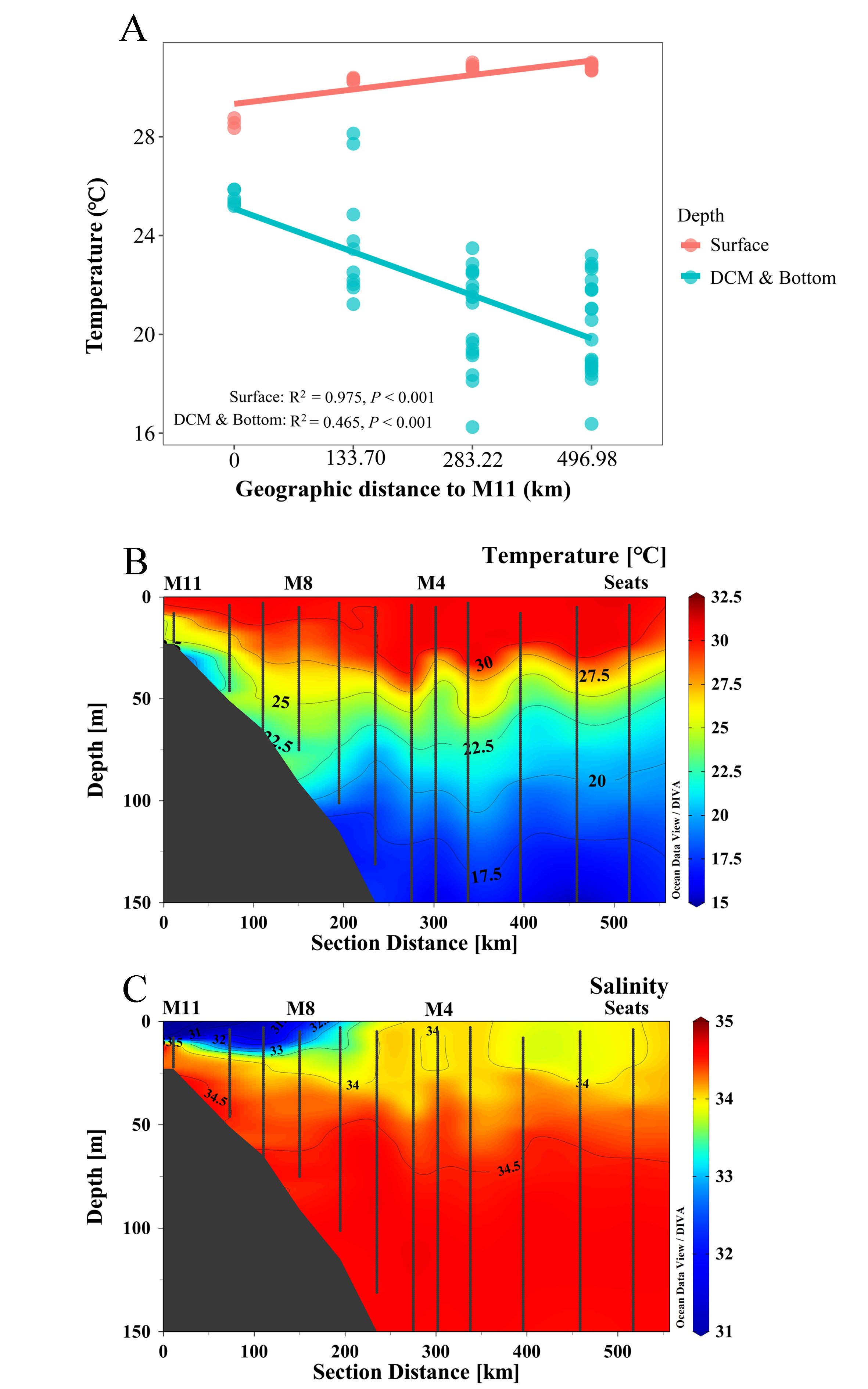

Supplement: FIG S5 [file msystems.00100-21-sf005.jpg]
