# Supplementary material for: Protistan-Bacterial Microbiota Exhibit Stronger Species Sorting and Greater Network Connectivity Offshore than Nearshore across a Coast-to-Basin Continuum
Source: mSystems. 2021 Oct 12;6(5):e00100-21. doi: 10.1128/mSystems.00100-21 (PMC8510552; doi:10.1128/mSystems.00100-21)
Supplement: TABLE S1 [file msystems.00100-21-st001.docx]

**Table S1.** Summary statistics of sequencing data in the present study.

|  | Protist | Bacteria |
| --- | --- | --- |
| Total raw reads | 16,260,974 | 20,766,004 |
| Reads after merging and quality control | 8,221,747 | 11,691,855 |
| Total reads after removal of singleton and chimera | 7,137,691 | 10,323,871 |
| Total reads after removal of non-protist/non-bacteria OTUs | 6,969,492 | 9,057,358 |
| Total reads after normalization | 3,491,208 | 2,706,984 |
| Total OTUs after normalization | 6,532 | 5,274 |
