# Supplementary material for: Protistan-Bacterial Microbiota Exhibit Stronger Species Sorting and Greater Network Connectivity Offshore than Nearshore across a Coast-to-Basin Continuum
Source: mSystems. 2021 Oct 12;6(5):e00100-21. doi: 10.1128/mSystems.00100-21 (PMC8510552; doi:10.1128/mSystems.00100-21)
Supplement: TABLE S2 [file msystems.00100-21-st002.docx]

**Table S2**. Multiple regression on dissimilarity matrices (MRM) results showed the percentage of variance in community composition of protist and bacteria explained by environmental variables. Values in bold indicate a significant correlation (p < 0.05). Ex.Var. (%), percentage of variance explained by environmental variables; Cum. (%), cumulative percentage of variance explained.

| Variable | Ex. Var. (%) | p | Cum. (%) |
| --- | --- | --- | --- |
| Protist |  |  |  |
| Temperature | **57.0** | 0.001 | 57.0 |
| Dissolved oxygen | **20.8** | 0.001 | 66.5 |
| Bacterial abundance | **20.4** | 0.001 | 72.4 |
| PNF 2-5 μm | **5.5** | 0.001 | 74.6 |
| Salinity | **17** | 0.001 | 74.6 |
| HNF 2-5 μm | **4.6** | 0.001 | 75.5 |
| Viral abundance | **5.5** | 0.001 | 75.8 |
| Chl *a* | **8.3** | 0.001 | 76.6 |
| HNF 5-20 μm | **1.1** | 0.014 | 76.8 |
| Bacteria |  |  |  |
| Temperature | **67.6** | 0.001 | 67.6 |
| Dissolved oxygen | **18.9** | 0.001 | 75.1 |
| Salinity | **19.4** | 0.001 | 81.6 |
| PNF2-5 μm | **4.7** | 0.001 | 82.0 |
| Chl *a* | **6.9** | 0.001 | 82.4 |
| HNF 5-20 μm | **1.0** | 0.012 | 82.5 |
