# Supplementary material for: Protistan-Bacterial Microbiota Exhibit Stronger Species Sorting and Greater Network Connectivity Offshore than Nearshore across a Coast-to-Basin Continuum
Source: mSystems. 2021 Oct 12;6(5):e00100-21. doi: 10.1128/mSystems.00100-21 (PMC8510552; doi:10.1128/mSystems.00100-21)
Supplement: TABLE S3 [file msystems.00100-21-st003.docx]

**Table S3.** Variation partitioning results showing the contribution of environmental ([E]) and spatial ([S]) factors.

|  | [S] | | [E] | | Selection/Dispersal limitation |
| --- | --- | --- | --- | --- | --- |
|  | var | p | var | p |  |
| Protist | 0.067 | 0.001 | 0.086 | 0.001 | 1.284 |
| Bacteria | 0.057 | 0.001 | 0.078 | 0.001 | 1.368 |
