# Supplementary material for: Protistan-Bacterial Microbiota Exhibit Stronger Species Sorting and Greater Network Connectivity Offshore than Nearshore across a Coast-to-Basin Continuum
Source: mSystems. 2021 Oct 12;6(5):e00100-21. doi: 10.1128/mSystems.00100-21 (PMC8510552; doi:10.1128/mSystems.00100-21)
Supplement: TABLE S4 [file msystems.00100-21-st004.docx]

**Table S4**. Sampling information of waters collected from the South China Sea.

| Sampling station | Longitude (°E) | Latitude (°N) | Sampling date | Sampling time | Sampling depth (m) | Bottom  depth (m) |  |
| --- | --- | --- | --- | --- | --- | --- | --- |
| M11 | 113.63 | 21.89 | 2019.6.29 | 0:00 | 5 | 23 | |
| M11 | 113.63 | 21.89 | 2019.6.29 | 0:00 | 15 | 23 | |
| M11 | 113.63 | 21.89 | 2019.6.29 | 0:00 | 25 | 23 | |
| M11 | 113.63 | 21.89 | 2019.6.29 | 6:00 | 5 | 23 | |
| M11 | 113.63 | 21.89 | 2019.6.29 | 6:00 | 10 | 23 | |
| M11 | 113.63 | 21.89 | 2019.6.29 | 6:00 | 25 | 23 | |
| M11 | 113.63 | 21.89 | 2019.6.29 | 12:00 | 5 | 23 | |
| M11 | 113.63 | 21.89 | 2019.6.29 | 12:00 | 10 | 23 | |
| M11 | 113.63 | 21.89 | 2019.6.29 | 12:00 | 23 | 23 | |
| M8 | 114.50 | 21.00 | 2019.6.27 | 12:00 | 5 | 91 | |
| M8 | 114.50 | 21.00 | 2019.6.27 | 12:00 | 25 | 91 | |
| M8 | 114.50 | 21.00 | 2019.6.27 | 12:00 | 75 | 91 | |
| M8 | 114.50 | 21.00 | 2019.6.27 | 18:00 | 5 | 91 | |
| M8 | 114.50 | 21.00 | 2019.6.27 | 18:00 | 40 | 91 | |
| M8 | 114.50 | 21.00 | 2019.6.27 | 18:00 | 75 | 91 | |
| M8 | 114.50 | 21.00 | 2019.6.28 | 0:00 | 5 | 91 | |
| M8 | 114.50 | 21.00 | 2019.6.28 | 0:00 | 60 | 91 | |
| M8 | 114.50 | 21.00 | 2019.6.28 | 0:00 | 75 | 91 | |
| M8 | 114.50 | 21.00 | 2019.6.28 | 6:00 | 5 | 91 | |
| M8 | 114.50 | 21.00 | 2019.6.28 | 6:00 | 55 | 91 | |
| M8 | 114.50 | 21.00 | 2019.6.28 | 6:00 | 75 | 91 | |
| M8 | 114.50 | 21.00 | 2019.6.28 | 12:00 | 5 | 91 | |
| M8 | 114.50 | 21.00 | 2019.6.28 | 12:00 | 58 | 91 | |
| M8 | 114.50 | 21.00 | 2019.6.28 | 12:00 | 75 | 91 | |
| M4 | 115.12 | 19.75 | 2019.6.24 | 12:00 | 5 | 1645 | |
| M4 | 115.12 | 19.75 | 2019.6.24 | 12:00 | 75 | 1645 | |
| M4 | 115.12 | 19.75 | 2019.6.24 | 12:00 | 100 | 1645 | |
| M4 | 115.12 | 19.75 | 2019.6.24 | 18:00 | 5 | 1645 | |
| M4 | 115.12 | 19.75 | 2019.6.24 | 18:00 | 75 | 1645 | |
| M4 | 115.12 | 19.75 | 2019.6.24 | 18:00 | 150 | 1645 | |
| M4 | 115.12 | 19.75 | 2019.6.25 | 0:00 | 5 | 1645 | |
| M4 | 115.12 | 19.75 | 2019.6.25 | 0:00 | 75 | 1645 | |
| M4 | 115.12 | 19.75 | 2019.6.25 | 0:00 | 100 | 1645 | |
| M4 | 115.12 | 19.75 | 2019.6.25 | 6:00 | 5 | 1645 | |
| M4 | 115.12 | 19.75 | 2019.6.25 | 6:00 | 75 | 1645 | |
| M4 | 115.12 | 19.75 | 2019.6.25 | 6:00 | 100 | 1645 | |
| M4 | 115.12 | 19.75 | 2019.6.25 | 12:00 | 5 | 1645 | |
| M4 | 115.12 | 19.75 | 2019.6.25 | 12:00 | 75 | 1645 | |
| M4 | 115.12 | 19.75 | 2019.6.25 | 12:00 | 100 | 1645 | |
| M4 | 115.12 | 19.75 | 2019.6.25 | 18:00 | 5 | 1645 | |
| M4 | 115.12 | 19.75 | 2019.6.25 | 18:00 | 75 | 1645 | |
| M4 | 115.12 | 19.75 | 2019.6.25 | 18:00 | 100 | 1645 | |
| M4 | 115.12 | 19.75 | 2019.6.26 | 0:00 | 5 | 1645 | |
| M4 | 115.12 | 19.75 | 2019.6.26 | 0:00 | 75 | 1645 | |
| M4 | 115.12 | 19.75 | 2019.6.26 | 0:00 | 100 | 1645 | |
| M4 | 115.12 | 19.75 | 2019.6.26 | 6:00 | 5 | 1645 | |
| M4 | 115.12 | 19.75 | 2019.6.26 | 6:00 | 75 | 1645 | |
| M4 | 115.12 | 19.75 | 2019.6.26 | 6:00 | 100 | 1645 | |
| M4 | 115.12 | 19.75 | 2019.6.26 | 12:00 | 5 | 1645 | |
| M4 | 115.12 | 19.75 | 2019.6.26 | 12:00 | 75 | 1645 | |
| M4 | 115.12 | 19.75 | 2019.6.26 | 12:00 | 100 | 1645 | |
| Seats | 116.00 | 18.00 | 2019.6.20 | 0:00 | 5 | 3907 | |
| Seats | 116.00 | 18.00 | 2019.6.20 | 0:00 | 75 | 3907 | |
| Seats | 116.00 | 18.00 | 2019.6.20 | 0:00 | 100 | 3907 | |
| Seats | 116.00 | 18.00 | 2019.6.20 | 6:00 | 5 | 3907 | |
| Seats | 116.00 | 18.00 | 2019.6.20 | 6:00 | 75 | 3907 | |
| Seats | 116.00 | 18.00 | 2019.6.20 | 6:00 | 100 | 3907 | |
| Seats | 116.00 | 18.00 | 2019.6.20 | 12:00 | 0 | 3907 | |
| Seats | 116.00 | 18.00 | 2019.6.20 | 12:00 | 65 | 3907 | |
| Seats | 116.00 | 18.00 | 2019.6.20 | 12:00 | 100 | 3907 | |
| Seats | 116.00 | 18.00 | 2019.6.20 | 18:00 | 0 | 3907 | |
| Seats | 116.00 | 18.00 | 2019.6.20 | 18:00 | 75 | 3907 | |
| Seats | 116.00 | 18.00 | 2019.6.20 | 18:00 | 100 | 3907 | |
| Seats | 116.00 | 18.00 | 2019.6.21 | 0:00 | 5 | 3907 | |
| Seats | 116.00 | 18.00 | 2019.6.21 | 0:00 | 65 | 3907 | |
| Seats | 116.00 | 18.00 | 2019.6.21 | 0:00 | 100 | 3907 | |
| Seats | 116.00 | 18.00 | 2019.6.21 | 6:00 | 5 | 3907 | |
| Seats | 116.00 | 18.00 | 2019.6.21 | 6:00 | 70 | 3907 | |
| Seats | 116.00 | 18.00 | 2019.6.21 | 6:00 | 100 | 3907 | |
| Seats | 116.00 | 18.00 | 2019.6.21 | 12:00 | 5 | 3907 | |
| Seats | 116.00 | 18.00 | 2019.6.21 | 12:00 | 65 | 3907 | |
| Seats | 116.00 | 18.00 | 2019.6.21 | 12:00 | 100 | 3907 | |
| Seats | 116.00 | 18.00 | 2019.6.21 | 18:00 | 5 | 3907 | |
| Seats | 116.00 | 18.00 | 2019.6.21 | 18:00 | 65 | 3907 | |
| Seats | 116.00 | 18.00 | 2019.6.21 | 18:00 | 100 | 3907 | |
| Seats | 116.00 | 18.00 | 2019.6.22 | 0:00 | 5 | 3907 | |
| Seats | 116.00 | 18.00 | 2019.6.22 | 0:00 | 65 | 3907 | |
| Seats | 116.00 | 18.00 | 2019.6.22 | 0:00 | 100 | 3907 | |
| Seats | 116.00 | 18.00 | 2019.6.22 | 6:00 | 5 | 3907 | |
| Seats | 116.00 | 18.00 | 2019.6.22 | 6:00 | 65 | 3907 | |
| Seats | 116.00 | 18.00 | 2019.6.22 | 6:00 | 100 | 3907 | |
| Seats | 116.00 | 18.00 | 2019.6.22 | 12:00 | 5 | 3907 | |
| Seats | 116.00 | 18.00 | 2019.6.22 | 12:00 | 65 | 3907 | |
| Seats | 116.00 | 18.00 | 2019.6.22 | 12:00 | 100 | 3907 | |
